# Mineralized tissue loss at the femoral ACL enthesis in young male ACL‐injured patients

**DOI:** 10.1002/jeo2.70106

**Published:** 2025-01-28

**Authors:** Mélanie L. Beaulieu, Yuchen Wang, Stephen H. Schlecht, James A. Ashton‐Miller, Edward M. Wojtys

**Affiliations:** ^1^ Department of Orthopaedic Surgery University of Michigan Ann Arbor Michigan USA; ^2^ Department of Orthopaedic Surgery Indiana University School of Medicine Indianapolis Indiana USA; ^3^ Department of Mechanical Engineering University of Michigan Ann Arbor Michigan USA; ^4^ Department of Biomedical Engineering University of Michigan Ann Arbor Michigan USA

**Keywords:** ACL reconstruction, bone health, graft integration, mineralized matrix, sex differences

## Abstract

**Purpose:**

Primary anterior cruciate ligament (ACL) reconstruction graft failure remains a significant health concern in young patients. Despite the high incidence of poor graft integration in these patients and the resulting high failure rate, little consideration has been given to the quality of the bone into which the graft is anchored at reconstruction. Therefore, we investigated post ACL injury mineralized tissue changes in the ACL femoral entheses of young males and compared them to changes previously reported for young females.

**Methods:**

ACL femoral entheses and adjacent bone specimens were harvested from the injured knees of 51 young males during primary ACL reconstructive surgery and from 10 non‐injured male cadaveric donors. The specimens were imaged via nano‐computed tomography and analyzed for volumetric bone mineral density (vBMD) and architectural changes.

**Results:**

Male femoral ACL explant specimens had significantly lower cortical vBMD (*p* < 0.001), lower relative bone volume (BV/TV, *p* = 0.027) and greater cortical bone porosity (Ct.Po, *p* = 0.027) but similar trabecular bone parameters (*p*'s > 0.05) to those of control specimens from male cadaveric donors. Cortical and trabecular bone loss increased significantly with time from ACL injury to reconstructive surgery (*p*'s < 0.05). While cortical loss occurred in both males and females, significant trabecular loss occurred only in females (*p* = 0.009).

**Conclusion:**

Femoral entheseal bone loss occurs in males following ACL injury. This bone loss increases with time following ACL injury, with cortical bone loss occurring sooner after injury than trabecular bone loss. The effects of ACL injury and time from injury to surgery on trabecular bone microarchitecture differed between male and female patients.

**Level of Evidence:**

N/A.

**Abbreviations**
ACL: anterior cruciate ligament
ANCOVA: analysis of covariance
ANOVA: analysis of variance
BMD: bone mineral density
BV/TV: relative bone volume (bone volume/total volume)
CF: calcified fibrocartilage
CT: computed topography
Ct.BV/TV: relative cortical bone volume
Ct.Po: cortical bone porosity
Ct.vBMD: volumetric cortical bone mineral density
DXA: dual‐energy X‐ray absorptiometry
I: injured
NI: non‐injured
PBS: phosphate‐buffered saline
Tb.BV/TV: relative trabecular bone volume
Tb.Sp: trabecular separation
Tb.Th: trabecular thickness
Tb.vBMD: volumetric trabecular bone mineral density
vBMD: volumetric bone mineral density
VOI: volume of interest

## INTRODUCTION

Between 9% and 18% of young patients (<18–21 years) and up to 28% of males under 18 years of age [42] receiving a primary anterior cruciate ligament (ACL) reconstruction will experience graft failure within five years [29, 42, 43]. Revision ACL reconstruction following graft failure is a major health concern because the results of this procedure are inferior to those of primary ACL reconstruction [44]. The most common reason for failure is a combination of factors, including biological factors [12, 19, 21, 22, 39, 45] such as insufficient osteogenic and ligamentous integration of the graft [46]. However, the quality of the bone into which the graft is anchored at primary reconstruction has received little consideration. Therefore, it is imperative to better understand how the mineralized matrices that anchor the native ligament, and subsequently the graft material, change following primary ACL injury.

Our overarching hypothesis was that primary graft failure is in part linked to graft fixation into a subpar post‐injury mineralized matrix, which then compromises graft osseointegration. It is well known that clinically measured bone mineral density (BMD) within the distal femur and proximal tibia, derived via dual‐energy X‐ray absorptiometry (DXA), drastically decreases following initial ACL injury [7, 24, 25, 27, 28, 30, 31]. However, this two‐dimensional low‐resolution BMD measurement cannot reveal degenerative bone changes occurring at the localized level of the ACL enthesis or provide specific information regarding the structural, compositional and mechanical integrity of the mineralized matrix. Filling this gap in knowledge is integral to furthering our understanding of why some primary ACL grafts fail and thus reduce the incidence of these types of graft failure. An important contribution toward bridging this gap was made recently by Patton et al. [35], who reported significant bone loss, especially in the cortical bone, within the femoral entheses of young female patients who underwent primary ACL reconstructive surgery. Ahn et al. [2] reverse‐translated these findings to an in vivo female mouse partial ACL injury model that confirmed cortical bone loss at the ACL femoral enthesis following injury. They also showed that this bone loss was not confined to the bone adjacent to the enthesis but instead extended to the entire condylar bone region. They hypothesized that such bone loss may be driven by inflammation, as opposed to altered loading of the femoral bone post‐injury (i.e., no tensile and shear loads applied by the ruptured ACL; altered knee mechanics following injury). Regardless of the mechanism(s) of post‐ACL injury bone changes, it remains unknown whether similar bone loss also occurs in age‐matched male patients and whether sex differences exist.

How bone loss at the ACL femoral enthesis changes over time following injury also remains unknown. Understanding this relationship is important, as it may inform the optimal timing for ACL reconstructive surgery. For example, operating within a specific timeframe, before further bone loss, could optimize osseointegration of the graft. The majority of ACL reconstructions are typically performed within the first three months following injury, a period that corresponds with the inflammation, regeneration and proliferation phases of the injured ligament [32]. However, a recent large‐scale study using the Danish Knee Ligament Reconstruction Registry reported a lower risk of revision ACL reconstructive surgery when primary surgery was delayed >3 or >6 months than when surgeries were performed <3 or <6 months post‐injury, respectively [23]. Therefore, it is also important to examine how ACL entheseal bone quality changes over time from injury to ACL injury to reconstructive surgery in young male patients.

The purpose of this study was to investigate post‐ACL injury changes in mineralized tissue in the ACL femoral entheses of young male patients and the effects of time from injury to ACL reconstructive surgery on these changes. Additionally, we aimed to compare these changes in young male patients with those previously reported in young female patients [35] by performing a secondary analysis of published female data [35]. We hypothesized that males would demonstrate significant losses in entheseal calcified fibrocartilage (CF) and cortical bone, compared with age‐matched non‐injured (NI) male controls and that this bone loss would be associated with the time from injury to ACL reconstructive surgery. We also tested the null hypothesis that there would be no significant difference in the mineralized matrices of the ACL femoral entheses post‐injury between males and females.

## MATERIALS AND METHODS

### Study design, sample populations, specimen extraction and preparation

In this retrospective case‐control study, the ACL femoral enthesis and adjacent bone (termed the ‘femoral ACL explant’) were extracted from the injured knee of 51 consecutive male patients during primary ACL reconstructive surgery and from 11 NI, unembalmed knees harvested from 10 male donors. The University of Michigan Institutional Review Board approved the use of deidentified patient and cadaveric donor tissues under an exempt status designation.

#### Patient group

**Figure 1:**
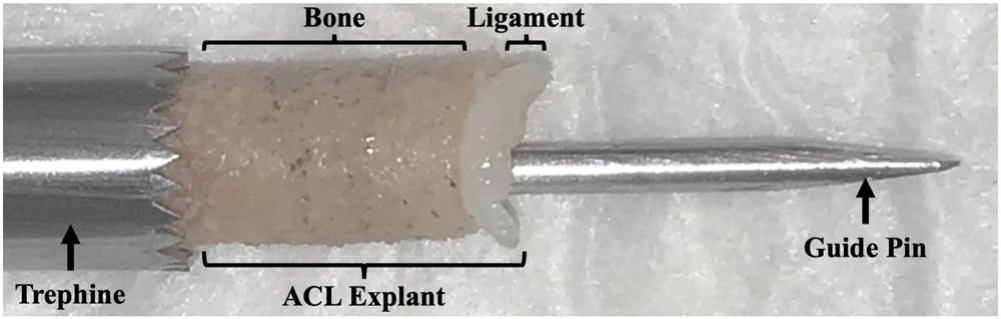
View of the 10‐mm steel trephine (on the left) and its guide pin (on the right) used in the extraction procedure in both patients and cadaveric donors. A sample femoral ACL explant specimen is also shown. ACL, anterior cruciate ligament. Reproduced from Chen et al [8] with permission from the authors.

The criteria used to include patients and their femoral ACL explants and the reasons for their exclusion are presented in Table 1. Patients were, on average, 19.3 ± 2.7 years of age, ranging from 14 to 25 years, at the time of ACL reconstructive surgery. Young male patients were selected to allow the data from their femoral ACL explant specimens to be combined with and compared to a published female patient data set [35] while matching the sample size and age range of those female patients. This is pertinent because young individuals are six times more likely to suffer a primary ACL graft failure within five years of surgery than older individuals [43]. Upon extraction, specimens were deidentified and age, sex, height, weight, activity at the time of ACL injury and time from injury to ACL reconstructive surgery were recorded. As previously described for the female specimens [35], a single surgeon (Author Initials) used a 10‐mm diameter steel trephine to remove ~50% of the entire native enthesis via standard ‘outside‐in’ surgical practices. Therefore, the femoral ACL explant specimens were 10 mm in diameter and approximately 6 mm in length and consisted of the remaining ligament tissue, the enthesis and the adjacent cortical and trabecular bone (Figure 1). Owing to the tissue extraction technique, each explant specimen also contained a 2.5‐mm diameter guide‐pin hole directly through its centre. The specimens were stored at 4°C in 1× phosphate‐buffered saline (PBS) upon extraction and imaged via nano‐computed tomography (CT) within 72 h.

**Table 1:** Inclusion criteria and reasons for exclusion for male patients and their femoral ACL explant specimens.

Inclusion criteria	Reasons for exclusion (*n* a)
Undergoing primary ACL reconstructive surgery performed by a single surgeon (EMW) within a maximum of 72 weeks^b^ of ACL injury	Undergoing revision ACL reconstructive surgery (*n* = 13) Undergoing surgery more than 72 weeks after ACL injury (*n* = 15)
Age ≤25 years	Age >25 years (*n* = 58)
Intact femoral ACL explant specimen was removed during reconstructive surgery	Pediatric patients (*n* = 3) Specimen was not intact upon removal (*n* = 5)
Nano‐CT imaging was acquired within 72 h	NanoCT imaging was not performed within this timeframe because staff and/or scanners were not available (*n* = 24)

Abbreviations: ACL, anterior cruciate ligament; CT, computed tomography.

^a^ Some patients were excluded for more than one reason.

^b^ This time delay between injury and surgery was selected to be consistent with the published work performed with female patients [35]. This allowed us to combine and compare the male patient data set with the published female patient data set.

^c^ In pediatric patients, we use a different surgical technique. It does not allow for the removal of femoral ACL explant specimens.

#### Control group

Knee specimens from which the control femoral ACL explant specimens were extracted were acquired from Gift of Life Michigan, Science Care and MedCure. The donors were, on average, 27.3 ± 8.1 years of age, ranging from 15 to 37 years, at the time of death. Acquiring specimens from donors with those demographics allowed us to balance the need to age‐match with our patient groups and the female control group [35] with the difficulty of acquiring specimens from young donors. The knee specimens, which were free of prior surgery, joint degeneration and any other visual indication of poor ACL enthesis and/or bone health, were stored in a freezer at −20°C until femoral ACL explant specimen extraction. The control femoral ACL explant specimens were extracted via the same methods as those used to extract the patient specimens. The control specimens were also stored at 4°C in 1× PBS upon extraction and imaged with nano‐CT within 72 h.

### Nano‐CT scanning preparation and acquisition

As previously described for the female specimens [35], all femoral ACL explant specimens were scanned at high resolution (14 μm voxel size) with a nanotom‐M CT system (phoenix|x‐ray, GE Sensing and Inspection Technologies, GmbH) using the following acquisition parameters: 70 kV, 300 μA, 34 min, 5000 ms exposure time, 1000 images, 0.012 in. aluminium filter. For imaging, the specimens were placed in a 5‐mL polypropylene scintillation vial, surrounded by polyurethane foam to prevent movement, and saturated in 1× PBS to maintain tissue hydration. To allow the grey values to be converted to Hounsfield units for bone mineral density quantification purposes, we included a calibration phantom containing air, water and a hydroxyapatite mimicker (1.69 mg/cc; Gammex) in each nano‐CT scan. The image volumes were reconstructed using datos|x reconstruction software (phoenix|x‐ray, GE Sensing and Inspection Technologies, GmbH).

### Nano‐CT image analysis

Prior to analysis, each femoral ACL explant was reoriented along the anteroposterior and mediolateral anatomical axes. The grey values in each reoriented image were converted to Hounsfield units using the calibration phantom, as previously described [35]. The cortical bone and the trabecular bone were manually segmented into two volumes of interest (VOIs). The guide pinhole volume, which was slightly expanded to include bone debris from drilling, was subtracted from the VOIs. The examination of trabecular bone in all samples was standardized to a thickness of 3.5 mm measured from the most inferior portion of the cortical matrix. The true volumetric bone mineral density (vBMD), relative bone volume (bone volume/total volume, BV/TV) and porosity (Ct.Po) were measured for the cortical VOI. The trabecular VOI parameters were vBMD, BV/TV, trabecular thickness (Tb.Th) and trabecular separation (Tb.Sp). The volumetric analyses were carried out via Dragonfly software (version 2022.2 for Windows 11. Comet Technologies Canada Inc.; software available at https://www.theobjects.com/dragonfly).

### Statistical analyses

The cortical and trabecular bone parameter data from the femoral ACL explants harvested from both the left and right knees of one of the control cadaveric donors were averaged [35]. The remaining explants were harvested from unpaired knees; therefore, 10 individual data points per bone parameter were used for our control group. To compare our results from the male femoral ACL explants to those previously published from female explants [35], we employed the same statistical analyses as those described in Patton et al. [35]. Briefly, to examine the effects of ACL injury on cortical and trabecular bone parameters, we used Welch's *t* test to compare the bone parameter data between patient and control ACL explant specimens. To examine the effect of the time delay between ACL injury and reconstructive surgery on the various bone parameters, we employed two approaches: (1) linear regression analysis with the predictor variable ‘time delay’ as a continuous variable using the patient specimen data and (2) Welch's *t* test with the independent variable ‘time delay’ grouped as NI, 1–7, 8–11, 12–16 and 17+ weeks. Finally, to examine the effect of age on the bone parameters, a multivariate linear regression approach with the predictors ‘age’, ‘time’ and their interaction (age*time delay) was used. The predictors ‘age’ and ‘time delay’ were mean centred to reduce multicollinearity problems that can arise when including predictors and their product terms in a regression.

In addition to the analyses performed with the female explant data in Patton et al. [35], we conducted a series of Welch's *t* tests with the independent variable ‘time delay’ grouped as NI, ≤3 months (≤13 weeks) and ≥6 months (≥26 weeks) post‐injury. To compare the mineralized tissue changes in ACL femoral entheses from male patients with those previously reported in young female patients [35], we combined the cortical and trabecular bone data of male patients and NI control specimens reported herein with those of a published female patient data set [35]. With this combined data set, we ran additional statistical analyses starting with one‐way analyses of variance (ANOVAs) to determine whether differences in age, weight and height existed between the sexes.

To determine whether patient sex moderates the effect of ACL injury on cortical and trabecular bone parameters, we examined the ‘sex‐by‐group’ interaction term of a series of 2 (sex) by 2 (group) analyses of covariance (ANCOVAs). We included age and height as covariates in these analyses, as well as in the subsequent analyses, to account for sex differences in patient/donor age and body size (see ‘Results’). Although sex differences were also observed in patient/donor weight, weight was not included as a covariate because of its correlation with height (see ‘Results’) and its lower correlations with the bone parameters than height (see ‘Results’) and because height explained more of the variance in the bone parameters than weight did when both covariates were included in preliminary regression models (data not presented). Including both variables could have introduced multicollinearity and unnecessarily reduced the degrees of freedom available for testing hypotheses, thereby diluting the statistical power of the analyses. In the case of a significant ‘sex‐by‐group’ interaction, post hoc analyses were conducted that assessed group differences within each sex separately with one‐way ANCOVAs.

To determine whether the effect of the time delay between ACL injury and reconstructive surgery on the various bone parameters was different between males and females, we examined the ‘sex‐by‐time’ interaction term of: (1) a series of multiple linear regressions with the predictor variables ‘time delay’ (as a continuous variable and mean centred), ‘sex’, ‘sex‐by‐time delay’, ‘age’ and ‘height’ and the cortical or trabecular bone parameter of interest as the outcome variable; (2) a series of 5 (time delay) by 2 (sex) ANCOVAs with the independent variable ‘time delay’ grouped as NI, 1–7, 8–11, 12–16 and 17+ weeks while accounting for the covariates ‘age’ and ‘height’; and (3) a series of 3 (time delay) by 2 (sex) ANCOVAs with the independent variable ‘time delay’ grouped as NI, ≤3 and ≥6 months post‐injury, also accounting for ‘age’ and ‘height’.

Finally, to determine whether the effect of patient age on the bone parameters differed by patient sex while accounting for the injury‐to‐surgery time delay, we examined the ‘sex‐by‐age’ interaction term of a series of multiple linear regressions with the predictor variables ‘age’, ‘time delay’, ‘age‐by‐time delay’, ‘sex‐by‐age’ and ‘height’. In these regressions, the mean‐centred values of ‘age’ and ‘time delay’, including their product terms, were used. All analyses were performed using SPSS (version 29). An *α* of 0.05 was used for all the statistical analyses to determine statistical significance.

## RESULTS

### Sample patient population

Nearly one third (31%) of the male patients injured their ACLs while playing football/flag football. Other prevalent activities during which ACL injuries occurred were basketball (18%), soccer (16%), skiing/snowboarding (10%) and lacrosse (10%). The remaining patients sustained their injury during a fall (4%), wrestling (4%), beach spikeball (2%), softball (2%) or trampolining (2%). Most (63%) primary ACL reconstructive surgeries occurred within 3 months post‐injury, whereas 20% occurred 6 months or longer post‐injury. On average, reconstructive surgery occurred 15.9 ± 15.2 weeks following injury, ranging from 2 to 70 weeks with a median of 9 weeks.

### Male ACL‐injured patients have femoral ACL entheseal cortical bone loss but not entheseal trabecular bone loss following ACL injury

**Figure 2:**
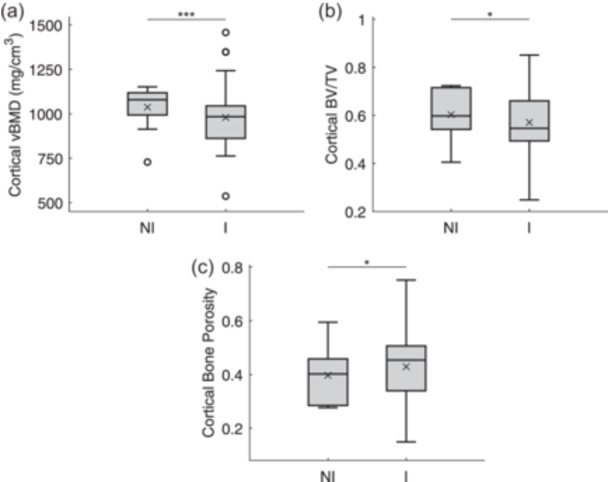
Box‐and‐whisker plots of the cortical bone parameters for the ACL femoral explant specimens from male non‐injured (NI) cadaveric donors and injured (I) patients: (a) volumetric bone mineral density (vBMD), (b) relative bone volume (BV/TV) and (c) bone porosity (Ct.Po). The box represents the first and third quartiles bisected by the median value. The lines extending below and above the box represent the minimum and maximum values, respectively, with the exception of the presence of potential outliers, each represented by a circle. The mean is represented by an ‘×’. Statistically significant differences between groups are represented by a horizontal line (*p < 0.05, **p < 0.01, ***p < 0.001). ACL, anterior cruciate ligament.

Compared with male NI control specimens, femoral ACL explant specimens from male ACL‐injured patients had significantly lower cortical vBMD (*p* < 0.001) and BV/TV (*p* = 0.027) and significantly greater cortical bone porosity (*p* = 0.027) (Figure 2). No significant differences in the trabecular bone parameters were detected between the injured and NI specimens (Tb.vBMD, *p* = 0.417; Tb.BV/TV, *p* = 0.398, Tb.Th, *p* = 0.146; Tb.Sp, *p* = 0.905).

### Both cortical and trabecular bone quality decreases as time from ACL injury to reconstructive surgery increases in male patients

**Figure 3:**
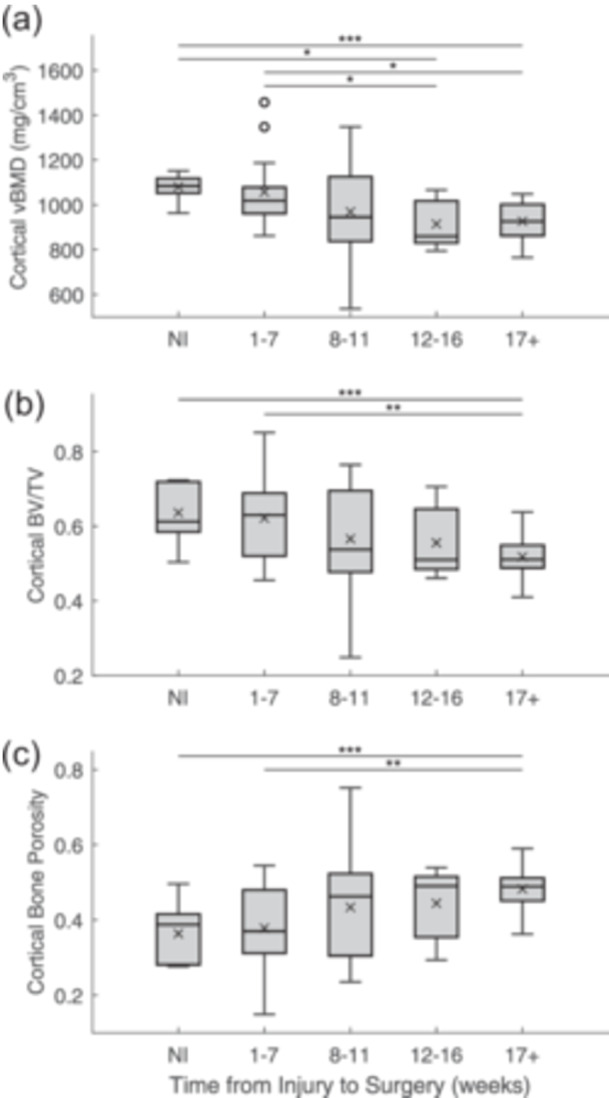
Box‐and‐whisker plots of the cortical bone parameters for the ACL femoral explant specimens from male non‐injured (NI) cadaveric donors and male patients who underwent ACL reconstructive surgery within 1–7, 8–11, 12–16 weeks and more than 17 weeks of injury: (a) volumetric bone mineral density (vBMD), (b) relative bone volume (BV/TV) and (c) bone porosity (Ct.Po). The box represents the first and third quartiles bisected by the median value. The lines extending below and above the box represent the minimum and maximum values, respectively, with the exception of the presence of potential outliers, each represented by a circle. The mean is represented by an ‘×’. Statistically significant differences between groups are represented by a horizontal line (*p < 0.05, **p < 0.01, ***p < 0.001).

**Figure 4:**
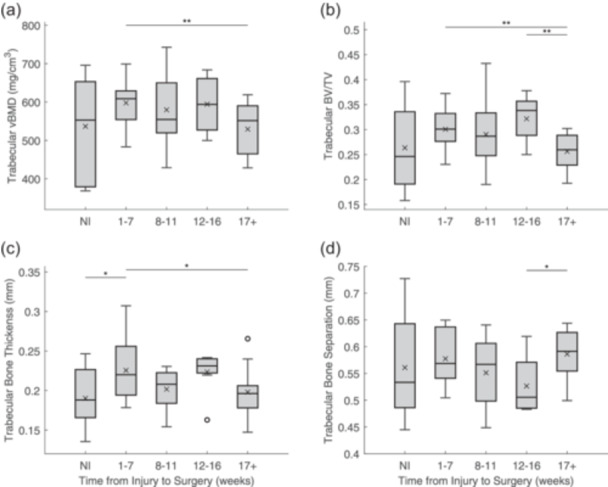
Box‐and‐whisker plots of the trabecular bone parameters for the ACL femoral explant specimens from male non‐injured (NI) cadaveric donors and male patients who underwent ACL reconstructive surgery within 1–7, 8–11, 12–16 weeks and more than 17 weeks of injury: (a) volumetric bone mineral density (vBMD), (b) relative bone volume (BV/TV), (c) trabecular thickness (Tb.Th) and (d) trabecular separation (Tb.Sp). The mean is represented by an ‘×’. Statistically significant differences between groups are represented by a horizontal line (*p < 0.05, **p < 0.01, ***p < 0.001). ACL, anterior cruciate ligament.

According to our linear regression analyses, there was no significant association between any of the cortical bone parameters and the time delay between ACL injury and reconstructive surgery (Ct.vBMD: *r* = 0.138, *p* = 0.341; Ct.BV/TV: *r* = 0.194, *p* = 0.177; Ct.Po: *r* = 0.194, *p* = 0.177). On the other hand, as time from injury to surgery increased, trabecular vBMD (*r* = 0.322, *p* = 0.021), BV/TV (*r* = 0.315, *p* = 0.024) and Tb.Th (*r* = 0.300, *p* = 0.033) significantly decreased. No significant association between time from injury to surgery and Tb.Sp was found (*r* = 0.112, *p* = 0.434). We took a closer look at the effect of the injury‐to‐surgery time delay on femoral entheseal bone quality by comparing groups of time delays. Cortical vBMD was significantly lower at 12‐16 weeks and at 17+ weeks than in the NI group (NI vs. 12–16 weeks: *p* = 0.05; NI vs. 17+ weeks: *p* < 0.001) and at 1–7 weeks (1–7 vs. 12–16 weeks: *p* = 0.038; 1–7 vs. 17+ weeks: *p* = 0.028) (Figure 3a). Similarly, cortical vBMD was significantly lower at ≤3 and ≥6 months post‐injury than it was in the NI group (NI vs. ≤3 mos: *p* = 0.043; NI vs. ≥6 mos: *p* < 0.001; Table 2). However, no significant difference was found between patients who had surgery within 3 months of injury and those who waited 6 months or more (*p *= 0.414; Table 2). In addition, the cortical BV/TV was significantly lower and Ct.Po was significantly greater at 17+ weeks than in the NI group (NI vs. 17+ weeks: Ct.BV/TV, *p* < 0.001; Ct.Po, *p* < 0.001) and at 1–7 weeks (1–7 vs. 17+ weeks: Ct.BV/TV, *p* = 0.006; Ct.Po, *p* = 0.006) (Figure 3b,c). Similarly, cortical BV/TV was significantly lower and Ct.Po was significantly greater at ≥6 months post‐injury than in the NI group (Ct.BV/TV: *p* = 0.004; Ct.Po: *p* = 0.004; Table 2). With respect to the trabecular bone parameters, Tb.vBMD, Tb.BV/TV and Tb.Th were significantly lower at 17+ weeks post‐ACL injury than at 1–7 weeks (Tb.vBMD: *p* = 0.009; Tb.BV/TV: *p* = 0.010; Tb.Th: *p* = 0.038) (Figure 4a–c). Similarly, Tb.vBMD and Tb.BV/TV were significantly lower at ≥6 months than at ≤3 months post‐injury (Tb.vBMD: *p* = 0.021; Tb.BV/TV: *p* = 0.006; Table 2) Tb.BV/TV was significantly lower and Tb.Sp was significantly greater at 17+ weeks than at 12–16 weeks (Tb.BV/TV: *p* = 0.009; Tb.Sp: *p* = 0.033) (Figure 4b,d). Finally, Tb.Th was found to be significantly greater in the patients at 1–7 weeks post‐injury than in the control specimens in the NI group (*p* = 0.035) (Figure 4c).

**Table 2:** Mean ± one standard deviation, cortical and trabecular bone parameters of male non‐injured controls and male ACL‐injured patients according to time delay between injury and reconstructive surgery.

	Groups		
	Non‐injured	Patients ≤3 months	Patients ≥6 months
Ct.vBMD (mg/cm^3^)	1080.2 ± 56.6^a^, ^b^	997.5 ± 196.5^a^	962.6 ± 73.7^b^
Ct.BV/TV	0.636 ± 0.078^b^	0.584 ± 0.128	0.538 ± 0.049^b^
Ct.Po	0.364 ± 0.078^b^	0.416 ± 0.128	0.462 ± 0.049^b^
Tb.vBMD (mg/cm^3^)	536.7 ± 130.8	586.2 ± 78.3^c^	520.8 ± 68.5^c^
Tb.BV/TV	0.264 ± 0.085	0.295 ± 0.052^c^	0.250 ± 0.038^c^
Tb.Th (mm)	0.190 ± 0.038	0.214 ± 0.032	0.194 ± 0.031
Tb.Sp (mm)	0.561 ± 0.093	0.566 ± 0.057	0.583 ± 0.051

Abbreviations: ACL, anterior cruciate ligament; Ct.BV/TV, relative cortical bone volume; Ct.Po, cortical bone porosity; Ct.vBMD, volumetric cortical bone mineral density; Tb.BV/TV, relative trabecular bone volume; Tb.Sp, trabecular separation; Tb.Th, trabecular thickness; Tb.vBMD, volumetric trabecular bone mineral density.

^a^ Statistically significant difference between non‐injured control group and ≤3 months post‐injury patient group.

^b^ Statistically significant difference between non‐injured control group and ≥6 months post‐injury patient group.

^c^ Statistically significant difference between ≤3 and ≥6 months post‐injury patient groups.

### Relative trabecular bone volume (BV/TV) significantly decreases as male patient age increases, accounting for time from ACL injury to reconstructive surgery

**Figure 5:**
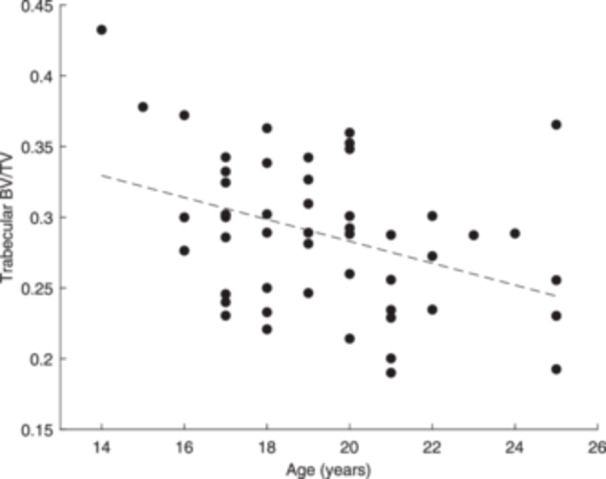
Scatterplot showing the significant association between relative trabecular bone volume (Tb.BV/TV) and patient age.

Age was not significantly associated with any of the cortical bone parameters, accounting for the time from ACL injury to reconstructive surgery (Table 3). For the trabecular bone parameters, only Tb.BV/TV was found to be significantly predicted by age when controlling for time from injury to surgery. As age increased, Tb.BV/TV significantly decreased (Figure 5). However, the effect of age on Tb.BV/TV did not differ by time from injury to surgery, as indicated by the non‐significant ‘age × time’ interaction term (Table 3).

**Table 3:** Results of multivariate linear regression modelling of the effects of male patient age, time from injury to ACL reconstructive surgery, and their interaction on cortical and trabecular bone parameters.

	Overall model statistics		Predictor statistics					
			Age		Time		Age*Time	
	*r*	*p*	*β* a	*p*	*β* a	*p*	*β* a	*p*
Ct.vBMD	0.272	0.310	0.238	0.133	−0.193	0.319	−0.049	0.786
Ct.BV/TV	0.197	0.606	0.022	0.893	−0.225	0.257	0.039	0.831
Ct.Po	0.197	0.606	−0.022	0.893	0.225	0.257	−0.039	0.831
Tb.vBMD	0.372	0.070	−0.115	0.446	−0.152	0.412	−0.221	0.202
Tb.BV/TV	**0.434**	**0.019**	**−0.330**	**0.027**	−0.149	0.407	−0.079	0.635
Tb.Th	0.315	0.174	−0.027	0.863	−0.219	0.249	−0.123	0.484
Tb.Sp	0.237	0.434	0.161	0.295	−0.076	0.692	0.221	0.223

*Note*: Bold denotes statistical significance.

Abbreviations: ACL, anterior cruciate ligament; Ct.BV/TV, relative cortical bone volume; Ct.Po, cortical bone porosity; Ct.vBMD, volumetric cortical bone mineral density; Tb.BV/TV, relative trabecular bone volume; Tb.Sp, trabecular separation; Tb.Th, trabecular thickness; Tb.vBMD, volumetric trabecular bone mineral density.

^a^ Standardized coefficient.

### Sex differences in patient/donor age and donor size: Evidence for including covariates in analyses examining sex differences in post‐injury femoral entheseal bone changes

Male patients and donors (patients: age, 19.3 ± 2.7 years; height, 1.79 ± 0.08 m; weight, 83.7 ± 14.1 kg | donors: age, 27.3 ± 8.1 years; height, 1.74 ± 0.08 m; weight, 67.1 ± 11.1 kg) were significantly older (*p* = 0.008), taller (*p* < 0.001) and heavier (*p* < 0.001) than female patients and donors (patients: age, 17.5 ± 2.8 years; height, 1.66 ± 0.07 m; weight, 68.2 ± 13.0 kg | donors: age, 25.3 ± 6.4 years; height, 1.63 ± 0.04 m; weight, 71.0 ± 11.5 kg). Patient/donor weight was significantly correlated with patient/donor height (*r* = 0.518, *p* < 0.001). Overall, the measures of BMD and bone microarchitecture were correlated to a greater degree with patient/donor height (Ct.vBMD: *r* = 0.537, *p* < 0.001; Ct.BV/TV: *r* = −0.149, *p* = 0.108; Ct.Po: *r* = 0.149, *p* = 0.108; Tb.vBMD: *r* = 0.618, *p* < 0.001; Tb.BV/TV: *r* = −0.060, *p* = 0.523; Tb.Th: *r* = 0.314, *p* < 0.001; Tb.Sp: *r* = 0.227, *p* = 0.014) than with patient/donor weight (Ct.vBMD: *r* = 0.273, *p* = 0.003; Ct.BV/TV: *r* = −0.203, *p* = 0.028; Ct.Po: *r* = 0.203, *p* = 0.028; Tb.vBMD: *r* = 0.352, *p* < 0.001; Tb.BV/TV: *r* = −0.186, *p* = 0.046; Tb.Th: *r* = 0.094, *p* = 0.313; Tb.Sp: *r* = 0.195, *p* = 0.036).

### Effect of ACL injury on trabecular bone microarchitecture in ACL‐injured male patients differs from that in female patients, accounting for sex differences in patient/donor age and size

The results revealed that female patients had some entheseal trabecular bone loss but not male patients. In particular, a significant ‘group‐by‐sex’ interaction was found for trabecular bone thickness (*p* = 0.036) when controlling for sex differences in patient/donor height and age. Post hoc analyses revealed that femoral ACL explant specimens from female patients had significantly lower Tb.Th values (*p* = 0.009) than those from NI female controls. Moreover, Tb.Th was not significantly different between the male injured and NI specimens (*p* = 0.779). The effects of ACL injury on the other trabecular bone parameters and on any of the cortical bone parameters were not significantly different between the male and female specimens (‘group‐by‐sex’ interaction: Tb.vBMD, *p* = 0.070; Tb.BV/TV, *p* = 0.059; Tb.Sp, *p* = 0.992; Ct.vBMD, *p* = 0.833, Ct.BV/TV, *p* = 0.338; Ct.Po, *p* = 0.338).

### Effect of time from ACL injury to reconstruction surgery on trabecular thickness in male patients differs from that in female patients, accounting for sex differences in patient/donor age and size

According to the ‘time‐by‐sex’ interaction term in our multiple linear regression models that controlled for the age and height of the ACL‐injured patients, sex did not moderate the associations between time from injury to reconstruction surgery and any of the cortical and trabecular bone parameters (Ct.vBMD: *p* = 0.594; Ct.BV/TV: *p* = 0.705; Ct.Po: *p* = 0.705; Tb.vBMD: *p* = 0.131; Tb.BV/TV: *p* = 0.336; Tb.Th: *p* = 0.476; Tb.Sp: *p* = 0.900). When the time delay between injury and surgery was categorized (NI, 1‐7, 8‐11, 12‐16, 17+ weeks), however, the relationship between time delay and Tb.Th was found to differ between male and female patients (‘time‐by‐sex’ interaction term: *p* = 0.013). Post hoc comparisons revealed that male patients had significantly lower Tb.Th values at 8–11 weeks post‐ACL injury than at 1–7 weeks post‐injury (*p* = 0.027). On the other hand, female patients at 12–16 weeks and at more than 17 weeks post‐injury had significantly lower Tb.Th values than the NI female control specimens (NI vs. 12–16 weeks: *p* = 0.032; NI vs. 17+ weeks: *p* = 0.025). Sex was not found to moderate the effect of time from injury to surgery (categorized as NI, 1–7, 8–11, 12–16 and 17+ weeks) on any of the other bone parameters (‘time‐by‐sex’ interaction term: Ct.vBMD, *p* = 0.352; Ct.BV/TV, *p* = 0.379; Ct.Po, *p* = 0.379; Tb.vBMD, *p* = 0.235; Tb.BV/TV, *p* = 0.083; Tb.Sp, *p* = 0.667). When time from injury to surgery was categorized as NI, ≤3 months, or ≥6 months, sex was not found to moderate the effect of this time delay on any of the bone parameters (‘time‐by‐sex’ interaction term: Ct.vBMD, *p* = 0.885; Ct.BV/TV, *p* = 0.490; Ct.Po, *p* = 0.490; Tb.vBMD, *p* = 0.130; Tb.BV/TV, *p* = 0.155; Tb.Th, *p* = 0.113; Tb.Sp, *p* = 0.950).

### Sex does not moderate the effect of patient age on bone quality, accounting for time from ACL injury to reconstructive surgery and patient size

According to the results from the multiple linear regression models, the associations between age and each cortical and trabecular bone parameter did not differ significantly between male and female patients. Specifically, the ‘age‐by‐sex’ interaction term was not statistically significant for any of the bone parameters (Ct.vBMD, *p* = 0.771; Ct.BV/TV, *p* = 0.994; Ct.Po, *p* = 0.994; Tb.vBMD, *p* = 0.219; Tb.BV/TV, *p* = 0.197; Tb.Th, *p* = 0.963; Tb.Sp, *p* = 0.421).

## DISCUSSION

Our results show that the entheseal cortical bone loss in these male patients post‐injury was consistent with the loss previously demonstrated in female patients [35]. The male patients, however, did not show entheseal trabecular bone loss, whereas the female patients had trabecular vBMD loss. Our analyses also revealed that both cortical and trabecular bone quality decreased in our male patients as the time delay between ACL injury and reconstructive surgery increased, with greater bone loss occurring six months or more after injury than within three months of injury.

Our findings support the hypothesis that male patients, like female patients [35], would demonstrate significant cortical bone loss in the femoral ACL entheseal tissue post‐ACL injury. In particular, we found that tissue specimens from male ACL‐injured patients, in comparison with those from NI control cadaveric donors, had significantly lower volumetric cortical bone mineral density and relative cortical bone volume (BV/TV), as well as greater cortical bone porosity; these effects of ACL injury on cortical bone did not differ between specimens from males and females after accounting for patient/donor age and height. Our results are consistent with the literature [2, 25]. In a longitudinal study of ACL‐injured male and female patients, Kroker et al. [25] reported cortical bone loss in the lateral femoral condyle of the injured knee. However, they reported that the layer of trabecular bone most adjacent to the cortical bone was mostly unaffected; only the vBMD decreased, similar to previous findings in females [35]. Additionally, a recently published small animal model demonstrated significant cortical bone loss but minimal trabecular bone loss at the ACL femoral enthesis following partial ACL rupture [2]. This study also suggests that our results of cortical bone loss may not be limited to the entheseal tissue but could extend to the entire knee joint. Ahn et al. [2] reported significant femoral and tibial bone loss; however, the magnitude of bone loss was similar between the entire condyles and the ACL entheses.

We propose several reasons why cortical bone was more affected than trabecular bone. First, cortical bone has a shorter bone resorption period and overall remodelling cycle than does trabecular bone [1, 14]. This implies that trabecular bone changes may occur later after injury. This is consistent with our finding that significant entheseal trabecular bone loss presented at ~4 months post‐injury compared with cortical bone loss at ~3 months post‐injury in our male patients. Since our patient's bone samples were extracted between 2 and 70 weeks post‐injury, with a median of 9 weeks, it is possible that trabecular bone loss was not captured due to the wide range of time from injury to surgery. Second, the trabecular bone data from the control specimens were much more variable than the cortical bone data. This may have contributed to the lack of significant differences in bone density and microarchitecture between patients and controls. Third, the literature suggests greater inflammatory potential in cortical bone than in trabecular bone [33]. The inflammatory response to ACL injury has been posited as a driving factor of bone loss in the injured knee [2, 25]. Finally, the small volume of our bone sample, which included only the shallowest portion of the trabecular bone (3.5 mm in depth), may have influenced the findings. As Kroker et al. [25] reported, this layer of trabecular bone in the lateral femoral condyle was mostly unaffected by ACL injury, whereas deeper layers of trabecular bone were negatively impacted.

Although our results do not elucidate the ‘why’ and ‘how’ of post‐injury bone loss, we suspect that it is driven by a combination of biological and, to a lesser degree, mechanical mechanisms. Mechanically, changes in knee loading [18], particularly the reduced loading of the bone adjacent to the ACL enthesis due to the absence of tensile and shear loads from the ruptured ACL, could lead to bone resorption. Although loading changes may contribute to bone adaptation, they are likely not the primary drivers of post‐injury femoral bone loss. First, unloading due to the absence of ACL loads occurs at the entheses, yet bone loss has been reported across the femoral condyles of the injured knee [2, 25], not solely at the femoral ACL enthesis. Second, while a decrease in tibiofemoral contact forces occurs post‐ACL injury [18], there is still substantial loading at the knee joint, making it unlikely that reduced loading alone is responsible for significant bone loss. Biologically, post‐injury inflammation is likely a major contributor to bone loss at the femoral ACL enthesis and throughout the femoral condyles [2, 37, 41]. After an ACL injury, the levels of pro‐inflammatory cytokines increase in the synovial fluid of the knee joint [6] and can remain elevated for more than a year [20], indicating a chronic inflammatory state. These cytokines promote catabolic processes in bone [13, 41], contributing to bone loss. Additionally, several factors, such as patient age and sex as well as the time delay from injury, mediate the effect of ACL injury on bone loss. We found a minimal effect of age on bone loss, likely due to the narrow age range (14–25 years) of the patients included in our study. We focused on young patients because most ACL injuries occur within this age group. However, Dauenhauer et al. [10] reported greater subchondral bone loss and increased bone resorption early post‐ACL injury in young mice (~20‐year‐old humans) than in older mice (~early‐old‐age humans). They speculated that their findings were largely due to age‐related effects on bone remodelling and inflammatory responses. The effects of sex and time delay on bone loss are discussed further below.

Regardless of the mechanism, bone loss at and near the ACL enthesis is a significant concern for the survival of ACL grafts. Our results suggest that many patients have bone tissue, particularly cortical bone tissue, that is not optimal for osseointegration of an ACL graft following reconstructive surgery. This is critical because of the importance of the cortical matrix surrounding the tunnel orifice, which is the optimal graft placement location in terms of graft healing and the restoration of mechanical function [17]. Aperture fixation is deemed essential for maintaining the long‐term functional stability of any graft [11]. Inadequate osseointegration at and near the tunnel orifice can lead to graft motion within the tunnel, such as the windshield wiper effect, which involves greater motion at the tunnel aperture [17], where we found significant cortical bone loss. Such graft motion can cause osteolysis, tunnel widening, abnormal graft function and eventual graft failure. Bone tunnel widening or enlargement is a well‐documented phenomenon in ACL reconstruction patients [47]. It may result from a complex interaction of biological, biomechanical and mechanical factors. Additionally, it appears to be more prevalent with hamstring autografts, which do not have the advantages of direct bone‐to‐bone contact and healing, as observed with bone‐patellar tendon‐bone (BTB) autografts [47]. A BTB graft allows strong fixation and early osteointegration in the tunnels. On the other hand, reliable fixation of the soft‐tissue hamstring graft at the tunnel orifice can be problematic [47]. These differences in bone‐graft integration between types of autografts, combined with the decreased quality of the bone surrounding the tunnel, may partly explain the increased failure rate of hamstring autografts [38]. Taken together, it is likely that bone quality and/or microarchitecture play a role in bone tunnel enlargement and, consequently, in the clinical outcomes of primary ACL reconstructive surgery and revision surgery. Longitudinal studies that track ACL graft survival rates in our patient cohort could elucidate this potential link between pre‐operative mineralized tissue quality, bone tunnel enlargement and graft function/survival.

The results from this study also support our hypothesis that significant mineralized tissue loss at the ACL femoral enthesis is associated with the time from injury to ACL reconstructive surgery in male ACL‐injured patients. We found that trabecular bone loss significantly increased as the time delay between injury and ACL reconstructive surgery also increased. What appears to drive this effect of time on trabecular bone changes is the significant trabecular bone loss occurring at more than 17 weeks ( ~4 months, Figure 4a–c), and more specifically at ≥6 months post‐injury (Table 2). These results suggest minimal trabecular bone loss in the first 3–4 months post‐injury. However, significant cortical bone loss at the ACL femoral enthesis occurred earlier, at 12–16 weeks (~3 months) post‐injury (Figure 3a, Table 2). A 10‐month longitudinal examination of femoral bone loss post‐injury revealed a non‐linear pattern of bone loss: accelerated loss after injury, with maximum loss occurring 31 weeks (~7 months) after injury on average, followed by a recovery phase [25]. We may have also observed this pattern had we included patients with an even longer delay between injury and surgery. Clearly, further research is needed. However, these post‐injury bone changes still raise the important question of whether greater consideration should be given to the surgical timing of ACL reconstructive surgery, beyond just its effect on cartilage and meniscal health [9, 15, 26, 34, 40] and thus its effect on ACL graft osseointegration. In terms of graft survival rates, a consensus has not been reached. There is evidence that delaying ACL reconstructive surgery is linked to lower successful clinical outcomes or a delay in reaching them, as well as increased rates of revision surgery [16]. Conversely, there is also evidence that delaying surgery for more than 3 or 6 months lowers a patient's risk of revision surgery [23].

Our findings partially reject our null hypothesis that post‐ACL injury entheseal bone loss does not differ between male and female patients. While there were no sex differences in ACL entheseal cortical bone changes following ACL injury, female patients did exhibit significant trabecular bone loss (i.e. lower trabecular bone thickness than NI female control specimens), which was not observed in male patients. These results differ from those reported by Patton et al. [35], who noted changes in only trabecular vBMD in the same cohort of female specimens. This discrepancy is most likely due to the different statistical models we used, which accounted for patient/donor age and height. Our results also differ from those reported by Ahn et al. [2], who reported no changes in entheseal trabecular vBMD or trabecular bone microarchitecture post‐ACL injury in female adolescent mice. Notably, the majority of the ACL injuries in the mice were partial tears. Additionally, changes in the entheseal bone were evaluated for up to 28 days post‐injury only.

The literature evaluating sex differences in post‐ACL injury femoral bone loss is sparse. In human studies, authors cite a lack of statistical power either for not investigating this factor [25] or for not finding sex to be a significant moderating factor [31]. However, in a mouse model, femoral bone loss post‐injury was greater in females than in males [5]. Sexual dimorphism in the inflammatory response to ACL injury may drive this sex difference in post‐injury bone loss [36]. Using an in silico model, Powell et al. [36] revealed that sex hormones in females may lead to a more pro‐inflammatory, catabolic environment in the knee joint, whereas males may have a more anti‐inflammatory environment. Given that a pro‐inflammatory environment can promote catabolic processes in bone [13, 41], as mentioned earlier, this may result in greater post‐injury bone loss in females than in males.

Our findings cannot speak directly to the effect of femoral entheseal bone quality on graft osteointegration, and subsequently graft survival rates. However, they do support our hypothesis that femoral entheseal bone is of lower quality in both female and male patients compared with control specimens, in terms of vBMD and its microarchitecture (i.e., lower relative cortical bone volume, and thus higher cortical bone porosity). This is especially true for the cortical bone into which the femoral tunnel is drilled and where the ACL graft is placed during reconstructive surgery. This catabolic activity within the entheseal footprint at the time of surgery may negatively affect ACL graft survival rates and increase the risk of osteolysis within the tunnel, and consequently, bone tunnel enlargement. This study, therefore, provides a foundation from which future research can investigate the survival rates of multiple graft types fixed at various points along the post‐injury continuum to determine how the bone status at the time of surgery correlates with long‐term graft adherence and integration. Combining this knowledge with other well‐studied risk factors will provide both physicians and patients with more confidence in the technique chosen for ACL reconstruction, the graft, its placement, and fixation while better ensuring that normal knee kinematics are restored for biomechanical stability.

The strengths and limitations of our study should be acknowledged. We present an analysis of a unique data set of nano‐CT images of the ACL femoral entheses extracted from 51 young male patients who underwent primary ACL reconstructive surgery. This is the first examination of the cortical and trabecular matrices of the tunnel where the ACL graft is fixed during reconstructive surgery in young (13–25 years) male patients and the first comparison to a cohort of female patients and control specimens [35]. Our high‐resolution imaging protocol (i.e., 14‐μm voxels) allowed us to investigate the three‐dimensional micro‐architecture of the cortical and trabecular bone separately, in contrast with previous work [7, 24, 27, 28, 30, 31], which mainly used DXA. Unfortunately, DXA only produces two‐dimensional low‐resolution measures of bone architecture, unable to quantify bone quality at the localized level of the ACL's entheses. On the other hand, our control specimens were not perfectly matched to our patient specimens for reasons inherent to cadaveric donors such as older age and an unknown premortem level of activity, both of which can affect bone quality. Given the impossibility of harvesting tissue from live NI controls, we argue that the strengths of our approach outweigh the weaknesses of using cadaveric tissue as control specimens. We successfully harvested control tissue from relatively young donors (i.e., 15–37 years) and we accounted for the remaining differences in patient/donor age and body size in our statistical models. Additionally, we acknowledge the small volume of our specimens (i.e., ~440 mm^3^ with a diameter of 10 mm). This may be problematic for two reasons: (1) it is difficult to rigorously analyze the trabecular bone; (2) it does not include the entire ACL femoral enthesis, which has a diameter of approximately 15 mm, on average, in adults [3] and is known to be heterogeneous in terms of the volume of calcified tissue [4]. Our tissue harvesting protocol, however, focused on removing the central part of the ACL femoral enthesis, thus providing a good representation of the entire enthesis and the adjacent bone into which the graft integrates post‐reconstructive surgery. We do acknowledge that there were slight differences in the location of the drilled tunnel in our patient sample because the surgeon must consider what is best for the patient. We also acknowledge that the entheseal bone that we analyzed for this study is the portion that is removed during surgery for graft placement; therefore, it is not the critical mineralized matrix that surrounds the tunnel where the graft will be placed and where osteointegration will occur. We argue, however, that our bone samples are closely representative of catabolic activity within the entheseal remnants given their proximity.

## CONCLUSIONS

Male ACL‐injured patients demonstrated significant cortical bone loss but minimal trabecular bone loss of the ACL femoral entheses between ACL injury and reconstructive surgery. As the time between ACL injury and reconstructive surgery increased, both cortical and trabecular bone quality decreased. Trabecular thickness was also found to decrease as patient age increased. Although cortical bone loss was similar in these male patients and in the female patients previously studied, there was a sex difference in trabecular bone loss. These findings suggest that the bone matrix, especially within the thin cortical matrix comprising the enthesis, may not always be optimal for osseointegration of an ACL graft, which could lead to abnormal graft function or failure.

## AUTHOR CONTRIBUTIONS

Mélanie L. Beaulieu, Stephen H. Schlecht, James A. Ashton‐Miller and Edward M. Wojtys were involved in project planning and data interpretation. Mélanie L. Beaulieu, Yuchen Wang, Stephen H. Schlecht and Edward M. Wojtys were involved in data collection, processing and/or analysis. All authors were involved in manuscript preparation and have read and approved the final submitted manuscript.

## CONFLICT OF INTEREST STATEMENT

The authors declare no conflicts of interest.

## ETHICS STATEMENT

The University of Michigan Institutional Review Board approved the use of patient and cadaveric tissues under an exempt status designation (HUM00097398).

## Data Availability

The data that support the findings of this study are available from the corresponding author upon reasonable request.

## References

[1] Agerbæk, M.O. , Eriksen, E.F. , Kragstrup, J. , Mosekilde, L. & Melsen, F. (1991) A reconstruction of the remodelling cycle in normal human cortical iliac bone. Bone and Mineral, 12(2), 101–112. Available from: 10.1016/0169-6009(91)90039-3 2015412

[2] Ahn, T. , Loflin, B.E. , Nguyen, N.B. , Miller, C.K. , Colglazier, K.A. , Wojtys, E.M. et al. (2023) Acute bone loss and infrapatellar fat pad fibrosis in the knee after an in vivo ACL injury in adolescent mice. The American Journal of Sports Medicine, 51(9), 2342–2356. Available from: 10.1177/03635465231180616 37366163 PMC10529334

[3] Beaulieu, M.L. , Carey, G.E. , Schlecht, S.H. , Wojtys, E.M. & Ashton‐Miller, J.A. (2015) Quantitative comparison of the microscopic anatomy of the human ACL femoral and tibial entheses. Journal of Orthopaedic Research, 33(12), 1811–1817. Available from: 10.1002/jor.22966 26134706 PMC4628572

[4] Beaulieu, M.L. , Carey, G.E. , Schlecht, S.H. , Wojtys, E.M. & Ashton‐Miller, J.A. (2016) On the heterogeneity of the femoral enthesis of the human ACL: microscopic anatomy and clinical implications. Journal of Experimental Orthopaedics, 3(1), 14. Available from: 10.1186/s40634-016-0050-8 27412665 PMC4943914

[5] Bergman, R.F. , Lammlin, L. , Junginger, L. , Farrell, E. , Goldman, S. , Darcy, R. et al. (2024) Sexual dimorphism of the synovial transcriptome underpins greater PTOA disease severity in male mice following joint injury. Osteoarthritis and Cartilage, 32(9), 1060–1073. Available from: 10.1016/j.joca.2023.07.012 37716404

[6] Bigoni, M. , Sacerdote, P. , Turati, M. , Franchi, S. , Gandolla, M. , Gaddi, D. et al. (2013) Acute and late changes in intraarticular cytokine levels following anterior cruciate ligament injury. Journal of Orthopaedic Research, 31(2), 315–321. Available from: 10.1002/jor.22208 22886741

[7] Boyd, S.K. , Matyas, J.R. , Wohl, G.R. , Kantzas, A. & Zernicke, R.F. (2000) Early regional adaptation of periarticular bone mineral density after anterior cruciate ligament injury. Journal of Applied Physiology, 89(6), 2359–2364. Available from: 10.1152/jappl.2000.89.6.2359 11090590

[8] Chen, J. , Kim, J. , Shao, W. , Schlecht, S.H. , Baek, S.Y. , Jones, A.K. et al. (2019) An anterior cruciate ligament failure mechanism. The American Journal of Sports Medicine, 47(9), 2067–2076. Available from: 10.1177/0363546519854450 31307223 PMC6905051

[9] Culvenor, A.G. , Eckstein, F. , Wirth, W. , Lohmander, L.S. & Frobell, R. (2019) Loss of patellofemoral cartilage thickness over 5 years following ACL injury depends on the initial treatment strategy: results from the KANON trial. British Journal of Sports Medicine, 53(18), 1168–1173. Available from: 10.1136/bjsports-2018-100167 30737199

[10] Dauenhauer, L.A. , Hislop, B.D. , Brahmachary, P. , Devine, C. , Gibbs, D. , June, R.K. et al. (2024) Aging alters the subchondral bone response 7 days after noninvasive traumatic joint injury in C57BL/6JN mice. Journal of Orthopaedic Research, 42, 2450–2460. Available from: 10.1002/jor.25921 38923623

[11] Deehan, D.J. & Cawston, T.E. (2005) The biology of integration of the anterior cruciate ligament. The Journal of Bone and Joint Surgery. British Volume, 87–B(7), 889–895. Available from: 10.1302/0301-620X.87B7.16038 15972898

[12] Dye, S.F. & Chew, M.H. (1993) Restoration of osseous homeostasis after anterior cruciate ligament reconstruction. The American Journal of Sports Medicine, 21(5), 748–750. Available from: 10.1177/036354659302100521 8238720

[13] Epsley, S. , Tadros, S. , Farid, A. , Kargilis, D. , Mehta, S. & Rajapakse, C.S. (2020) The effect of inflammation on bone. Frontiers in Physiology, 11, 511799. Available from: 10.3389/fphys.2020.511799 33584321 PMC7874051

[14] Eriksen, E.F. , Melsen, F. & Mosekilde, L. (1984) Reconstruction of the resorptive site in iliac trabecular bone: a kinetic model for bone resorption in 20 normal individuals. Metabolic Bone Disease and Related Research, 5(5), 235–242. Available from: 10.1016/0221-8747(84)90065-1 6493035

[15] Everhart, J.S. , Kirven, J.C. , Abouljoud, M.M. , DiBartola, A.C. , Kaeding, C.C. & Flanigan, D.C. (2019) Effect of delayed primary anterior cruciate ligament reconstruction on medial compartment cartilage and meniscal health. The American Journal of Sports Medicine, 47(8), 1816–1824. Available from: 10.1177/0363546519849695 31125273

[16] Forsythe, B. , Lu, Y. , Agarwalla, A. , Ezuma, C.O. , Patel, B.H. , Nwachukwu, B.U. et al. (2021) Delaying ACL reconstruction beyond 6 months from injury impacts likelihood for clinically significant outcome improvement. The Knee, 33, 290–297. Available from: 10.1016/j.knee.2021.10.010 34739960

[17] Fu, F.H. , Bennett, C.H. , Lattermann, C. & Ma, C.B. (1999) Current trends in anterior cruciate ligament reconstruction. The American Journal of Sports Medicine, 27(6), 821–830. Available from: 10.1177/03635465990270062501 10569374

[18] Gardinier, E.S. , Manal, K. , Buchanan, T.S. & Snyder‐Mackler, L. (2013) Altered loading in the injured knee after ACL rupture. Journal of Orthopaedic Research, 31(3), 458–464. Available from: 10.1002/jor.22249 23097309 PMC3553294

[19] Graf‘, B. & Uhr, F. (1988) Complications of intra‐articular anterior cruciate reconstruction. Clinics in Sports Medicine, 7(4), 835–848. Available from: 10.1016/S0278-5919(20)30889-9 3052885

[20] Harkey, M.S. , Luc, B.A. , Golightly, Y.M. , Thomas, A.C. , Driban, J.B. , Hackney, A.C. et al. (2015) Osteoarthritis‐related biomarkers following anterior cruciate ligament injury and reconstruction: a systematic review. Osteoarthritis and Cartilage, 23(1), 1–12. Available from: 10.1016/j.joca.2014.09.004 25219671

[21] Harner, C.D. , Irrgang, J.J. , Paul, J. , Dearwater, S. & Fu, F.H. (1992) Loss of motion after anterior cruciate ligament reconstruction. The American Journal of Sports Medicine, 20(5), 499–506. Available from: 10.1177/036354659202000503 1443315

[22] Jaureguito, J.W. & Paulos, L.E. (1996) Why grafts fail. Clinical Orthopaedics and Related Research, 325, 25–41. Available from: 10.1097/00003086-199604000-00005 8998884

[23] Jensen, H.A. , Nielsen, T.G. & Lind, M. (2024) Delaying anterior cruciate ligament reconstruction for more than 3 or 6 months results in lower risk of revision surgery. Journal of Orthopaedics and Traumatology, 25(1), 19. Available from: 10.1186/s10195-024-00759-1 38637340 PMC11026352

[24] Knurr, K.A. , Kliethermes, S.A. , Haack, C.R. , Olson, J.S. , Binkley, N.C. , Scerpella, T.A. et al. (2022) Changes in bone mineral density of the femur and tibia before injury to 2 years after anterior cruciate ligament reconstruction in Division I collegiate athletes. The American Journal of Sports Medicine, 50(9), 2410–2416. Available from: 10.1177/03635465221099456 35647798 PMC9703853

[25] Kroker, A. , Besler, B.A. , Bhatla, J.L. , Shtil, M. , Salat, P. , Mohtadi, N. et al. (2019) Longitudinal effects of acute anterior cruciate ligament tears on peri‐articular bone in human knees within the first year of injury. Journal of Orthopaedic Research, 37(11), 2325–2336. Available from: 10.1002/jor.24410 31283044

[26] Krutsch, W. , Zellner, J. , Baumann, F. , Pfeifer, C. , Nerlich, M. & Angele, P. (2017) Timing of anterior cruciate ligament reconstruction within the first year after trauma and its influence on treatment of cartilage and meniscus pathology. Knee Surgery, Sports Traumatology, Arthroscopy, 25(2), 418–425. Available from: 10.1007/s00167-015-3830-2 26475153

[27] Leppälä, J. , Kannus, P. , Natri, A. , Pasanen, M. , Sievänen, H. , Vuori, I. et al. (1999) Effect of anterior cruciate ligament injury of the knee on bone mineral density of the spine and affected lower extremity: a prospective one‐year follow‐up study. Calcified Tissue International, 64(4), 357–363. Available from: 10.1007/s002239900632 10089231

[28] Lui, P.P.Y. , Cheng, Y.Y. , Yung, S.H. , Hung, A.S.L. & Chan, K.M. (2012) A randomized controlled trial comparing bone mineral density changes of three different ACL reconstruction techniques. The Knee, 19(6), 779–785. Available from: 10.1016/j.knee.2012.02.005 22425308

[29] Maletis, G.B. , Chen, J. , Inacio, M.C.S. & Funahashi, T.T. (2016) Age‐related risk factors for revision anterior cruciate ligament reconstruction: a cohort study of 21,304 patients from the Kaiser Permanente Anterior Cruciate Ligament Registry. The American Journal of Sports Medicine, 44(2), 331–336. Available from: 10.1177/0363546515614813 26637284

[30] van Meer, B.L. , Waarsing, J.H. , van Eijsden, W.A. , Meuffels, D.E. , van Arkel, E.R.A. , Verhaar, J.A.N. et al. (2014) Bone mineral density changes in the knee following anterior cruciate ligament rupture. Osteoarthritis and Cartilage, 22(1), 154–161. Available from: 10.1016/j.joca.2013.11.005 24269632

[31] Mündermann, A. , Payer, N. , Felmet, G. & Riehle, H. (2015) Comparison of volumetric bone mineral density in the operated and contralateral knee after anterior cruciate ligament and reconstruction: a 1‐year follow‐up study using peripheral quantitative computed tomography. Journal of Orthopaedic Research, 33(12), 1804–1810. Available from: 10.1002/jor.22962 26123943

[32] Murray, M.M. , Martin, S.D. , Martin, T.L. & Spector, M. (2000) Histological changes in the human anterior cruciate ligament after rupture. The Journal of Bone and Joint Surgery. American Volume, 82–A(10), 1387–1397. Available from: 10.2106/00004623-200010000-00004 11057466

[33] Omar, O. , Suska, F. , Lennerås, M. , Zoric, N. , Svensson, S. , Hall, J. et al. (2011) The influence of bone type on the gene expression in normal bone and at the bone‐implant interface: experiments in animal model. Clinical Implant Dentistry and Related Research, 13(2), 146–156. Available from: 10.1111/j.1708-8208.2009.00195.x 19438950

[34] Papastergiou, S.G. , Koukoulias, N.E. , Mikalef, P. , Ziogas, E. & Voulgaropoulos, H. (2007) Meniscal tears in the ACL‐deficient knee: correlation between meniscal tears and the timing of ACL reconstruction. Knee Surgery, Sports Traumatology, Arthroscopy, 15(12), 1438–1444. Available from: 10.1007/s00167-007-0414-9 17899001

[35] Patton, D.M. , Ochocki, D.N. , Martin, C.T. , Casden, M. , Jepsen, K.J. , Ashton‐Miller, J.A. et al. (2022) State of the mineralized tissue comprising the femoral ACL enthesis in young women with an ACL failure. Journal of Orthopaedic Research, 40(4), 826–837. Available from: 10.1002/jor.25130 34191360 PMC8716678

[36] Powell, B. , Szleifer, I. & Dhaher, Y.Y. (2018) In silico study of principal sex hormone effects on post‐injury synovial inflammatory response. PLoS One, 13(12), e0209582. Available from: 10.1371/journal.pone.0209582 30596697 PMC6312367

[37] Redlich, K. & Smolen, J.S. (2012) Inflammatory bone loss: pathogenesis and therapeutic intervention. Nature Reviews Drug Discovery, 11(3), 234–250. Available from: 10.1038/nrd3669 22378270

[38] Samuelsen, B.T. , Webster, K.E. , Johnson, N.R. , Hewett, T.E. & Krych, A.J. (2017) Hamstring autograft versus patellar tendon autograft for ACL reconstruction: is there a difference in graft failure rate? A meta‐analysis of 47,613 patients. Clinical Orthopaedics & Related Research, 475(10), 2459–2468. Available from: 10.1007/s11999-017-5278-9 28205075 PMC5599382

[39] Shelbourne, K.D. , Wilckens, J.H. , Mollabashy, A. & DeCarlo, M. (1991) Arthrofibrosis in acute anterior cruciate ligament reconstruction. The effect of timing of reconstruction and rehabilitation. The American Journal of Sports Medicine, 19(4), 332–336. Available from: 10.1177/036354659101900402 1897645

[40] Taketomi, S. , Inui, H. , Yamagami, R. , Kawaguchi, K. , Nakazato, K. , Kono, K. et al. (2018) Surgical timing of anterior cruciate ligament reconstruction to prevent associated meniscal and cartilage lesions. Journal of Orthopaedic Science, 23(3), 546–551. Available from: 10.1016/j.jos.2018.02.006 29501276

[41] Terkawi, M.A. , Matsumae, G. , Shimizu, T. , Takahashi, D. , Kadoya, K. & Iwasaki, N. (2022) Interplay between inflammation and pathological bone resorption: insights into recent mechanisms and pathways in related diseases for future perspectives. International Journal of Molecular Sciences, 23(3), 1786. Available from: 10.3390/ijms23031786 35163708 PMC8836472

[42] Webster, K.E. & Feller, J.A. (2016) Exploring the high reinjury rate in younger patients undergoing anterior cruciate ligament reconstruction. The American Journal of Sports Medicine, 44(11), 2827–2832. Available from: 10.1177/0363546516651845 27390346

[43] Webster, K.E. , Feller, J.A. , Leigh, W.B. & Richmond, A.K. (2014) Younger patients are at increased risk for graft rupture and contralateral injury after anterior cruciate ligament reconstruction. The American Journal of Sports Medicine, 42(3), 641–647. Available from: 10.1177/0363546513517540 24451111

[44] Weiler, A. , Schmeling, A. , Stöhr, I. , Kääb, M.J. & Wagner, M. (2007) Primary versus single‐stage revision anterior cruciate ligament reconstruction using autologous hamstring tendon grafts: a prospective matched‐group analysis. The American Journal of Sports Medicine, 35(10), 1643–1652. Available from: 10.1177/0363546507303114 17575015

[45] Wetzler, M.J. , Bartolozzi, A.R. , Gillespie, M.J. , Rubenstein, D.L. , Ciccotti, M.G. & Miller, L.S. (1996) Revision anterior cruciate ligament reconstruction. Operative Techniques in Orthopaedics, 6(3), 181–189. Available from: 10.1016/S1048-6666(96)80018-8

[46] Wright, R.W. , Huston, L.J. , Spindler, K.P. , Dunn, W.R. , Haas, A.K. , Allen, C.R. et al. (2010) Descriptive epidemiology of the Multicenter ACL Revision Study (MARS) cohort. The American Journal of Sports Medicine, 38(10), 1979–1986. Available from: 10.1177/0363546510378645 20889962 PMC3655411

[47] Yue, L. , DeFroda, S.F. , Sullivan, K. , Garcia, D. & Owens, B.D. (2020) Mechanisms of bone tunnel enlargement following anterior cruciate ligament reconstruction. JBJS Reviews, 8(4), e0120. Available from: 10.2106/JBJS.RVW.19.00120 32539260

